# Host Molecules That Promote Pathophysiology of Ocular Herpes

**DOI:** 10.3389/fmicb.2022.818658

**Published:** 2022-01-25

**Authors:** Sajal Deea Shukla, Tibor Valyi-Nagy

**Affiliations:** ^1^Department of Pathology, College of Medicine, University of Illinois at Chicago, Chicago, IL, United States; ^2^Illinois Mathematics and Science Academy, Aurora, IL, United States

**Keywords:** herpes simplex virus type 1 (HSV-1), ocular infection, pathophysiology, pathogenesis, host determinants

## Abstract

Herpes simplex virus type-1 (HSV-1) is a human virus that causes lifelong infections in a large population worldwide. Recurrence of HSV-1 from latency in trigeminal ganglion (TG) is the trigger of the morbidities seen with this virus. In addition to causing fever blisters and cold sores, occasionally the virus can also cause corneal lesions resulting in blindness in untreated individuals. Several host cell proteins play important roles in HSV-1 infection of the eye. HSV-1 enters into the corneal epithelial cells *via* its interactions with cell surface receptors. In parallel, the Toll-like receptors sense viral invasion and activate defense mechanisms to fight the infection. New data shows that Optineurin, a host autophagy receptor is also activated to degrade viral particles. In contrast, activation of heparanase, a host enzyme, induces an immune-inflammatory response, which triggers pro-inflammatory and pro-angiogenic environment and ultimately results in many of the clinical features seen with HSV-1 infection of the cornea. Rarely, HSV-1 can also spread to the central nervous system causing serious diseases. In this review, we summarize the latest knowledge on host molecules that promote pathophysiological aspects of ocular herpes.

## Introduction

Herpes simplex virus-1 (HSV-1) is a double-stranded DNA virus, which belongs to the Alpha herpesvirus subfamily of herpesviruses (Whitley and Roizman, 2001). It is a nuclear replicating enveloped virus, which is often acquired during childhood after direct contact with infected skin lesions, saliva or other body fluids. Humans are its natural hosts but this virus with extreme host range can infect virtually all cell types and experimental animals (Karasneh and Shukla, 2011). In humans, it predominantly infects mucosal epithelia of the orofacial region including the eye and establishes lifelong latency in the neurons especially those present in the trigeminal ganglia (Whitley and Roizman, 2001; Farooq et al., 2010; Farooq and Shukla, 2012). During latency, HSV-1 remains dormant expressing the latency associated transcript (LAT) and possibly a small number of other viral proteins. While symptomatic reactivation is less common virtually all infected individuals tend to asymptomatically shed the reactivated virus ***via*** tears and saliva (Kaufman et al., 2005). Given its affinity to establish infection of the neurons, the virus is classified as a neurotropic virus. It is a ubiquitous virus infecting as high as 80% of the human population. HSV infections are largely asymptomatic, yet the infected individual can shed the virus independent of the occurrence of any obvious disease manifestations (Kaufman et al., 2005). The virus causes a wide range of symptomatic diseases but the lesions or ulcers on oral mucosa or lips (herpes labialis) are the most common manifestation of the viral infection. In addition, herpetic whitlow, herpes gladiatorum, eczema herpeticum and herpetic sycosis of the beard area as well herpetic eye infections are routinely reported. In extreme cases, an uncontrolled spread of primary or reactivated HSV-1 to central nervous system (CNS) can initiate a rapidly progressing and potentially fatal encephalitis in humans (Fraser and Valyi-Nagy, 1993).

## Virion Structure and Lifecycle

**FIGURE 1:**
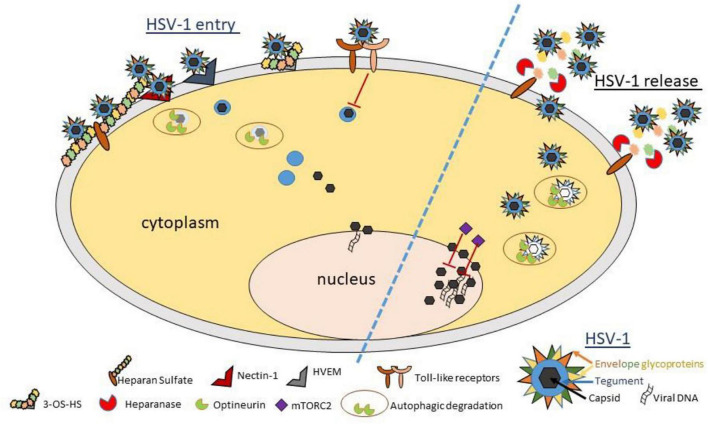
Schematic diagram and model showing important host proteins and their roles in pathophysiology of ocular herpes infection. HSV-1 entry into a corneal cell starts with the binding of viral glycoproteins gB and/or gC to cell surface heparan sulfate. Next, HSV-1 gD binds to one of its cognate receptors (Nectin-1, HVEM, and 3-OS HS) to start viral capsid penetration into the cytoplasm. Toll-like receptors sense viral invasion and activate intrinsic immune responses to control infection. A newly identified restriction factor, optineurin, reduces incoming or outgoing viral load by selective degradation of viral capsid and essential proteins *via* autophagy. In parallel, mTORC2 complex acts to reduce viral replication in the nucleus. In a pro-viral role, heparanase, a host enzyme is upregulated to facilitate HSV-1 release from the corneal cell by removal of cell surface HS. While the events summarized here do not describe the complete picture of viral invasion, they do highlight many new findings and interventional targets in context with HSV-1 infection of the cornea. A cartoon describing the essential structural components of a matured HSV-1 virion is shown (bottom right).

Herpesvirus family members share an identical structure. They are primarily composed of a lipid bilayer envelope that expresses about a dozen glycoproteins required for viral entry and cell-to-cell spread; a tegument, which mainly contains proteins including transcriptional regulators required for initial infection; a capsid, which contains a linear copy of the double stranded DNA genome (Karasneh and Shukla, 2011; Figure 1). Electron micrograph of the virions suggests about 200 nm spherical structure that contains an icosahedral capsid, which is about ∼125 nm in diameter (Dai and Zhou, 2018; Yuan et al., 2018). Spikes of glycoproteins protrude from the viral envelop. Many of the envelope glycoproteins are highly conserved among herpesviruses and proven to be essential for viral infectivity (Shukla and Spear, 2001). Viral DNA genome is about 150 kbp long, and it is known to encode for 80 or more genes (Whitley and Roizman, 2001). The HSV genes have high GC contents in the range of 67%. Tegument is a collection of nearly a dozen proteins, which play important roles in promoting viral transcription, reducing host transcription and disrupting the host antiviral response early during infection (Dai and Zhou, 2018).

## Infection of the Eye

Infection of the eye by HSV-1 is considered the most frequent cause of corneal blindness in the developed world including the United States (Farooq and Shukla, 2012). HSV infection of the conjunctiva, iris or the cornea is accompanied by an acute onset of pain. Blurring of vision, mostly arising from the inflammation of the local tissue, ensues shortly after the infection causing rapid vision issues and discomfort. Depending on the immune status of an individual (immune competent or compromised) virtually all types of ocular cells and tissues show susceptibility to HSV-1 infection. The most common manifestations of ocular HSV-1 infection include conjunctivitis, blepharitis, keratitis, iritis, uveitis, and retinitis (Lobo et al., 2019). HSV epithelial keratitis normally reported as dendritic ulcers can limit itself to the corneal epithelium. It is more common and easier to resolve *via* the use of topical and/or systemic antivirals such as Acyclovir or its analogs (Farooq and Shukla, 2012). Occasionally, HSV-1 can spread into the corneal stroma causing an immunoinflammatory condition called stromal keratitis. Stromal keratitis can sustain for long durations and cause severe vision loss due to scarring of the tissue. Similarly, HSV-1 associated cases of conjunctivitis, blepharitis, iritis, uveitis and retinitis are also reported (Lobo et al., 2019). Often the conditions can be resolved *via* use of antiviral chemotherapy and local or systemic corticosteroids to reduce inflammation (Liesegang, 1988, 2001).

## Expression of Herpes Simplex Virus-1 Entry Receptors in the Eye

Entry of HSV-1 into natural target cells such as the cells of the corneal epithelium and the innervating neurons is a multistep process that requires participation from many viral envelope glycoproteins as well as a few host entry receptors expressed on the cell surface (Farooq et al., 2010). Four out of a dozen or so different glycoproteins that are present on the viral envelope play essential roles during entry. These include glycoproteins gB, gD, gH, and gL (Madavaraju et al., 2021). HSV-1 mutants lacking any one of these glycoproteins fail to infect target cells. A fifth glycoprotein, called gC, also participates but it is not considered essential for entry (Spear, 2004). A simple understanding of their functions in viral entry comes from host cell receptors that they interact with. Most understood among these are the receptors for gB, gC, and gD. The former (gB and gC) are thought to make the initial contact with a target cell *via* their attachment or binding to heparan sulfate proteoglycans (HSPG) (Shukla and Spear, 2001). HSPG are expressed on the surface of virtually all mammalian cells (Sarrazin et al., 2011). The proteoglycans contain one or more negatively charged carbohydrate chains known as heparan sulfate (HS) that are covalently linked to a protein core. HS moieties make the initial charge-based interaction with HSV-1 glycoproteins gB and gC. Basically, they bind to positively charged motifs on these glycoproteins. The nature of protein core is likely to be important since downregulation of a couple of HSPG, such as syndecan-1 or syndecan-2 severely reduces HSV-1 entry (Bacsa et al., 2011). Interaction with HSPG is not essential for entry but loss of HS from cell surface, such as the one found in a mutant cell line, reduces HSV-1 entry down to ∼10% of normal levels (Shukla et al., 1999). HSPG interaction is not only important for the virion attachment to cells, but it is also needed for extracellular transport of the virions on cell membrane projections called filopodia. This transport phenomenon termed, viral surfing, enhances the efficacy of HSV-1 infection by allowing a targeted unidirectional movement of extracellular particles toward the body of the cell (Oh et al., 2010). It is considered an actin/myosin dependent transport phenomenon that has the potential to enhance infection at the site of a tissue injury where the leading edge of remaining cells generate a large amount of filopodia. After the initial attachment *via* gB and gC, gD is recruited to find its receptor. Binding of gD to one of its host cell receptors is considered essential for entry. The gD receptors (described below) include nectin-1, HVEM and 3-O sulfated HS (Spear et al., 2000; Figure 1). This key interaction with gD receptor allows the viral envelope to fuse with plasma membrane of the host cell, releasing the viral tegument proteins and the capsid into the cytoplasm. Alternatively, in corneal fibroblasts, a phagocytosis-like uptake can deliver the capsid into the cytoplasm (Clement et al., 2006). The capsid is then efficiently transported on the host cell microtubules for delivery to the nuclear membrane. Host cell motors such as dynein or kinesin participate in this intracellular transport of the capsid. The inbound transport of the capsid during entry is mediated by dynein, whereas the outbound (toward the plasma membrane for virion release) is performed by kinesin-1 (Banerjee et al., 2020; DuRaine and Johnson, 2021). Once the capsid reaches the nuclear membrane it uncoats and releases the viral DNA into the nucleus for replication. Delivery of the viral DNA into the nucleus also marks the end of the final step of HSV-1 entry.

## Herpes Simplex Virus-1 Entry Receptors and Their Expression Patterns in the Eye

All known gD receptors are expressed in the eye. Details on their abundance and expression patterns are provided below.

### Ocular Nectin-1

Nectin-1 is considered the most widely expressed entry receptor for HSV-1 in the eye (Valyi-Nagy et al., 2004). It belongs to the immunoglobulin superfamily, mediates cell-cell adhesion in cadherin-based adherens junctions, and acts as a receptor for herpes simplex virus (HSV) (Spear et al., 2000). In murine ocular tissues widespread expression of nectin-1 expression can be detected on cells of the conjunctiva, corneal epithelium as well as the endothelium, iris, ciliary body, lens epithelium, choroid, and the retina (Valyi-Nagy et al., 2004). Interestingly, fibroblasts especially those found in the corneal stroma and the sclera do not express nectin-1 (Tiwari et al., 2006). As a result, in the absence of detectable amounts of nectin-1, HVEM and 3-O sulfated HS (discussed below) are considered to be the key receptors for HSV-1 entry into the corneal stroma. Also was interesting that HSV-1 infection (lytic or latent) did not result in a loss of nectin-1 expression in any of the murine tissues examined (Valyi-Nagy et al., 2004). No loss in receptor expression during latency is an important finding since reactivated HSV-1 in the TG could potentially use the available receptors for traveling back to the corneal epithelium causing recurrent damage to the tissue. Nectin-1 is also expressed abundantly in murine brain indicating its potential use during cell-to-cell spread of HSV-1 from the eye to the brain (Shukla et al., 2006; Prandovszky et al., 2008).

### Herpes Virus Entry Mediator

Herpes virus entry mediator (HVEM) is the second most commonly expressed receptor for HSV-1 entry into the cells of the eye (Kovacs et al., 2009). Its normal function involves regulation of tissue inflammation *via* interaction with physiological ligands such as LIGHT and lymphotoxin alpha (Cai and Freeman, 2009). It is considered a molecular switch for inducing stimulatory or inhibitory immune signaling (Ware, 2009). Studies using immunohistochemistry show that it is easily detectable in the conjunctiva as well as the corneal epithelium and endothelium (Akhtar et al., 2008; Kovacs et al., 2009). However, it is less detectable and focally expressed in the corneal stroma. Interestingly, primary infection with HSV-1 causes increased expression of HVEM in the corneal epithelium and stroma (Kovacs et al., 2009). The nerves innervating the corneal stroma also seem to express detectable levels of HVEM (Kovacs et al., 2009). It is a major mediator for entry into the human conjunctival epithelial cells (Akhtar et al., 2008). Independent of its role as an entry receptor for the virus, HVEM plays an additional role in HSV-1 reactivation from latency. A recent study provides exciting new information that gD-independent immune modulating functions of HVEM can regulate HSV-1 latency and reactivation cycles (Tormanen et al., 2020). Thus, given its role in immune control of HSV-1 latency, it is possible that activation of NF-kappaB during HSV-1 infection may drive an upregulation of HVEM expression in certain tissues (Sciortino et al., 2008).

### 3-O Sulfated Heparan Sulfate

Heparan sulfate proteoglycans (HSPGs) are well expressed on the corneal surface as well as other tissues in the eye (Tanihara et al., 2002). In retinal tissues, HSPGs are expressed in the membrane of the retinal cells and secreted into the extracellular space of retinal tissues, contributing directly to the neural network formation required for vision processing. In certain situations, including immunocompromised patients HSV-1 virions can infect the retina as well (Cordero-Coma et al., 2007). Abundant expression of HSPGs are detected in corneal epithelium and the stroma. While HSPGs provide non-specific attachment sites for many different viruses and bacteria, 3-O Sulfated Heparan Sulfate (3-OS HS), which is a subdomain of HS generated after modification of the parent chain by 3-O sulfotransferases, is a well-established receptor for HSV-1 gD (Shukla et al., 1999). Its expression on the corneal surface was demonstrated by the binding of specific peptides that were generated against 3-OS HS. These peptides bind 3-OS HS on the murine corneal surface and block HSV-1 infection of the cornea (Tiwari et al., 2011). 3-OS HS is also expressed by the corneal stromal cells. Given the lack of specific antibodies against this subdomain of HS very little information exists about its expression in other ocular sites. Likewise, its significance in viral reactivation from latency remains poorly defined.

## Host Virulence Factors

It is relatively common that viral infections exploit host proteins for supporting their replication, growth and maturation. It is also common that host cells respond to infection by up-regulating certain proteins that have the potential to cause damage to infected cells and tissues. Discussed below are a few important proteins that play important roles in pathophysiology of HSV-1 infection of the eye.

### Proangiogenic Factors

Angiogenesis or neovascularization can be a hallmark of HSV-1 infection in the cornea. Healthy corneas are not vascularized, which improves clarity of vision. The so called “corneal angiogenic privilege” (Azar, 2006) is acquired because multiple mechanisms such as tight packaging of collagen lamellae, higher expression of anti-angiogenic factors and lower expression of pro-angiogenic proteins such as matrix metalloproteinases (MMPs) generate an environment that retards new blood vessel formation and promotes clear vision. In contrast, an increase in aberrant corneal vascularization including leaky or improperly formed vessels upon HSV-1 infection can result in the clouding of the cornea and loss of vision (Lobo et al., 2019). HSV-1 infection can cause an up-regulation of proangiogenic factors such as vascular endothelial growth factors (VEGF), fibroblast growth factor (FGF) and MMPs (Park et al., 2015). Evidence exist that VEGF-A production can be enhanced by an HSV-1 encoded immediate early transcription factor, infected cell protein 4 (ICP-4) (Wuest et al., 2011). In addition, HSV-1 infection causes VEGF-A release from its receptor bound inactive form (Suryawanshi et al., 2011). Both actions directly promote angiogenesis, endothelial cell proliferation as well as migration, and capillary tube formation. Similarly, expression and activity of FGF-2, which is a HS-binding peptide, is significantly enhanced upon HSV-1 infection of the cornea (Gurung et al., 2018). FGF2 and other structurally related polypeptides of FGF family are potent inducers of vasculature growth, endothelial cell survival as well as normal and pathological hematopoiesis (Qazi et al., 2009). FGF2 can promote corneal angiogenesis by providing proliferative signaling as well as regulating expression of other proangiogenic factors including VEGF and MMPs. The latter have been well studied in context with neutrophil-induced angiogenesis (Lee et al., 2002). Studies demonstrate that infiltrating neutrophils secrete MMP-9 which contributes to neovascularization of the cornea upon HSV-1 infection. A direct role for MMPs in HSV-1 infection is still debatable since it may depend on neutrophil signaling and complexity generated by the simultaneous release of other cytokines and growth factors. Presence of gamma delta T cells in the cornea during HVS-1 infection also supports angiogenesis *via* IL- 17A production that in turn induces a local spike in levels of VEGF (Suryawanshi et al., 2012). Somewhat indirectly, certain chemokines (e.g., CCL2, CXCL1, and CXCL2) induced during HSV-1 infection attract inflammatory cells (such as neutrophils) that contribute directly to new vessel formation (Giménez et al., 2013).

### Pro-inflammatory Factors

Cytokines such as IL-1β, TNFα, and IL-6 have been well documented as the key mediators of inflammation in the eye (Santacruz et al., 2015). During HSV-1 infection IL-1α, IL-6, IL-17, and IFN-γ as well as certain chemokines (e.g., MCP-1, MIP-1α, MIP-1β, and MIP-2) mediate proinflammatory conditions that promote neutrophil infiltration and corneal damage (Azher et al., 2017). Similarly, IL-17, a proinflammatory cytokine, is upregulated during infection and considered a key participant in the pathogenesis of herpetic lesion development (Suryawanshi et al., 2011; Amin et al., 2019). It signals tissue infiltration by neutrophils, supports neutrophil survival and locally drives cells to release harmful molecules such as oxyradicals and certain MMPs. MMP-9 is a known marker for corneal inflammation and a well-established sheddase for HSPGs such as Syndecans (Bollmann et al., 2021). Similar to MMPs, heparanase (HPSE), is also a well-studied HSPG sheddase (Oshima et al., 2021). It is expressed in cells of the cornea and very well studied for its unique enzymatic activity as an endo-β-D-glucuronidase in mammalian cells (Agelidis and Shukla, 2020). It is the only mammalian enzyme known to cleave HS moieties present on proteoglycans and promote their shedding from the surface. In that role it regulates the remodeling of the extracellular matrix (ECM). HPSE is synthesized in the endoplasmic reticulum as a 65 kDa proenzyme precursor that undergoes proteolytic cleavage to yield 50 and 8 kDa subunits that heterodimerize to form the active enzyme. The active enzyme is transported to the exterior of the cell where it binds its substrate HS to cause its cleavage. Studies have shown that it is upregulated late during HSV infection through the action of transcription factor, NF-kB (Hadigal et al., 2015). Infection-activated HPSE is translocated to the cell surface where it removes HS. Once HS, the attachment receptor for HSV-1, is removed from the cell surface, the newly produced progeny virions are released to the ECM without the risk of getting trapped by the mother cell, which in turn, contributes significantly to enhance viral release (Hadigal et al., 2015). Not only cultured cells but also infected murine corneas demonstrate an increase in HPSE expression upon HSV-1 infection. Knockdown of HPSE *in vivo* inhibits virus shedding. Since activated HPSE is a known contributor to inflammation and angiogenesis it was shown that it acts a molecular trigger to exacerbate the disease pathologies associated with HSV-1 infection of the eye (Agelidis et al., 2017). In human corneal epithelial cells, upregulation of HPSE shows dependence on NF-kB as well as HSV-1 infected cell protein 34.5 (γ34.5). Studies demonstrate that HPSE also could relocate to the nucleus to regulate β-catenin signaling (Koujah et al., 2021), promote release of nuclear HS-bound proangiogenic factors and regulate proinflammatory cytokine production. Its actions in the cell nucleus generate pro-inflammatory and pro-angiogenic conditions and help establish HPSE as a host virulence factor. In addition to directly promoting a toxic environment inside an HSV-1 infected cell, HPSE can also restrict cell-intrinsic defense mechanisms resulting in a hostile takeover of the host cell reprogramming by HSV-1 (Agelidis et al., 2021a). HPSE through its diverse yet well-regulated interactome can interfere with double-stranded DNA breakage response by blocking signal transduction between sensors and downstream mediators thereby reducing the cell’s ability to fight HSV-1 infection (Agelidis et al., 2021b). Quite in contrast, the HPSE knockout cells resist HSV-1 infection and use robust antiviral mechanisms to thwart infection.

## Corneal Tissue-Expressed Host Proteins That Prevent or Limit Herpes Simplex Virus Pathophysiology

At any given instance the corneal surfaces is exposed to many pathogens including bacteria and viruses. While the tear film itself does a good job of restricting access to the cornea several newly identified proteins expressed in the cornea also tend to limit the impact of infection either by activating the innate immune response or directly participating in the clearance of HSV-1 virions. Two major examples are discussed below.

### Toll-Like Receptors

These plasma membrane as well as endosomal membrane-expressed proteins are considered innate defense sensors against a variety of pathogens including HSV-1 (Finberg et al., 2005). The proteins belong to an evolutionarily conserved family of pattern recognition receptors that activate innate and adaptive immune pathways after sensing molecular patterns associated with various pathogens. Toll-like receptors (TLR) detect pathogen-associated molecular patterns (PAMP), which are invariant molecular structures found within pathogens including HSV-1 (Jin et al., 2007). TLR survey the plasma membrane and intracellular compartments for the presence of viral signatures such as viral genetic material and/or proteins. Once a signature is detected, TLR and their cytosolic receptors engage their downstream adaptor proteins to induce inflammatory cytokines and other effector molecules to fight off the infection and also, if required, cause death to the infected cells (Finberg et al., 2005). Mitochondrial antiviral-signaling protein (MAVS) and stimulator of IFN genes (STING) provide the cytosolic adaptor functions and process the signals generated after engagement with TLRs. Transcriptional activation of interferon regulatory factor-3 (IRF3) and -7 (IRF7) is a direct result of TLR activation, which in turn, induces the expression of IFN-α/β and other antiviral chemokines (Ma and He, 2014). Detection of HSV *via* TLRs also signals the assembly of inflammasomes and induction of proinflammatory cytokines. Currently about a dozen TLRs have been identified. Out of those, at least 6 TLRs (TLR1, 2, 3, 7, 8, and 9) may be involved in HSV–associated PAMP detection (Finberg et al., 2005; Jin et al., 2007). It is already known that TLR2 can uniquely recognize HSV-1 envelope glycoproteins and lipopeptides, whereas TLR9 recognizes CpG motifs present in HSV-1 genome. At the level of gene expression, mRNAs for virtually all known human TLRs in corneal epithelium can be detected by RT-PCR. Interestingly, all the TLR mRNA expression is upregulated in corneas with active HSV-1 infection. Among these TLR4, 8, and 9 showed the highest expression in HSV-1 infected human corneas (Jin et al., 2007). Given the significant increase in expression it is speculated that TLR4, 8, and 9 may also be involved in sensing of HSV-1 infection of the cornea. At the protein level, both healthy as well as HSV-1 infected corneas demonstrated Immunofluorescent staining for TLR2 and 9 expressions. Interestingly, TLR9 expression is less pronounced in healthy corneas, but it is significantly enhanced in corneas with active HSV-1 (Jin et al., 2007). Significant differences were not evident in TLR2 expression under healthy or infected conditions. Other TLRs have not been thoroughly examined for ocular tissue distribution and likewise, their precise roles in HSV-1 ocular pathogenesis remain poorly defined.

### Optineurin

Genetic screens have implicated Optineurin (OPTN), which is also known as optic neuropathy inducing protein, in many neurodegenerative diseases including primary open-angle glaucoma (Ying and Yue, 2012). The gene encoding the OPTN protein is well-expressed in the eye (Rezaie et al., 2007). Until recently the functional role(s) of OPTN in a viral disease were poorly defined. A recent study showed that OPTN can selectively restrict HSV-1 infection of the cornea and the brain (Ames et al., 2021). It was demonstrated that OPTN selectively targets HSV-1 tegument protein, VP16, and gB, which is essential for viral spread from cell-to-cell. The authors showed that VP16 and gB bind to OPTN and the protein complex is degraded by autophagy. When mice lacking the gene for the OPTN protein were infected with HSV-1 *via* the cornea, the majority of animals failed to survive the infection within 10 days of exposure to the virus. Quite interestingly the infected animals show significant cognitive decline. Using a novel object recognition test, the authors demonstrated that healthy animals either lacking OPTN gene or the controls with intact OPTN gene showed a similar preference to explore novel objects. However, after a month post ocular HSV-1 infection the controls but not the OPTN mutant animals maintained the same preference. Indicating a loss of cognition related to memory the mutant animals did not show any preference for novel objects. The authors also demonstrated that OPTN functions in the cornea are important for the recruitment of adaptive immunity and suppression of neuronal necroptosis. A major outcome of this study was that ocular HSV-1 infection can be lethal without OPTN as the virus can spread to the central nervous system (CNS) and cause encephalitis.

## Innate and Adaptive Immune Responses That Control Herpes Simplex Virus-1 Infection of the Eye

In addition to the newly discovered factors discussed above, several well established innate and adaptive immune mechanisms prevent viral replication and provide direct control over HSV-1 infection of the eye. The immune controls exist at primary infection sites such as the cornea or conjunctiva and within the TG, where they regulate latency and reactivation (Rolinski and Hus, 2014). It is the reactivation of HSV-1 from latency in TG that triggers the morbidities associated with this virus. The innate immune mechanisms that act almost immediately to control infection include production of type I interferons such as IFN-α and IFN-β by infected cells (Ma and He, 2014). These cytokines are important for controlling both lytic and latent HSV infections. Along the same line, IFN-γ helps drive the virus in latent state and acts directly to prevent reactivation (De Regge et al., 2010). Many innate immune cells such as dendritic cells (DC), macrophages, natural killer (NK) cells and mast cells (MC) protect the corneal tissue from primary as well as reactivated HSV-1 infection (Rolinski and Hus, 2014). The local population of DC, which is considered the major producer of type I IFNs, plays a major role in defense against HSV-1. In parallel, NK cells, which may also reside locally, produce antiviral cytokines, and specifically target HSV-1 infected cells for killing (Bigley, 2014). Local as well as infiltrating populations of macrophages also provide the initial as well as persistent defense against lytic and latent infections (Mott et al., 2007). Similarly, infiltrating neutrophils are often the most prominent innate immune cell type during HSV-1 infection of the cornea. However, their role in HSV-1 infection may be dubious as they may cause proinflammatory environment and local tissue damage (Azher et al., 2017). IL-17 with its high potency proinflammatory functions is initially released by innate immune cells such as γδ T cells (Suryawanshi et al., 2012). IL-17 may be a chemoattractant for neutrophil influx into the cornea. In addition to the innate immune cells, the adaptive immune cells such as HSV-1 specific CD4 + T and CD4 + T cells provide robust control against HSV-1 infection of the eye (Farooq and Shukla, 2012). The CD8 + T cells reduce infection and inhibit viral replication by lytic granule-dependent as well as IFN-γ-dependent effector mechanisms (Rolinski and Hus, 2014). These cells are thought to be effective in keeping the virus under latency and preventing its reactivation (Khanna et al., 2003). However, some recent findings downplay the significance of CD8 + T cells in the maintenance of latency (Mott et al., 2016). Similarly, virus specific CD4 + T cells may contribute to HSV-1 immune pathologies and lesion development in the cornea, but they are also beneficial and helpful in HSV-1 clearance from the primary site of infection and also, reducing the local viral load (Buela and Hendricks, 2015). A CD4 + T cell subtype called regulatory T cells (Treg) also contributes to the progression of herpes keratitis but its immunomodulatory role is still emerging and far from being well defined (Varanasi et al., 2017). Like any other viral infections, humoral immunity against HSV-1 is important but it is not effective in controlling the virus possibly because antibody access to the virions may be limited as they spread internally from cell to cell and usually not found in the blood, which is a common source of spread for many other viral diseases (Pollara et al., 2004).

## Herpes Simplex Virus-1 Encephalitis

Following primary infection of mucocutaneous sites including the cornea, HSV-1 uses axonal routes to reach the peripheral nervous system including the trigeminal ganglia for establishing latency. Often, the virus can also make its way to the CNS nervous system. As a result, HSV-1 genomic DNA can be found in the CNS of a subset of the adult human population (Fraser et al., 1981; Baringer and Pisani, 1994; Itzhaki et al., 1997). Despite the presence of HSV-1 DNA in the brain, any strong evidence of viral reactivation in the CNS remains elusive. For poorly defined reasons encephalitis develops in only a small minority of human cases involving primary and reactivated HSV-1 infections. Still HSV-1 remains the most common cause of sporadic encephalitis in the United States. The molecular mechanisms by which HSV-1 damages the nervous system require additional studies. While local immune system including aberrant release of proinflammatory cytokines such as TNF-α certainly has a role, specific mediators such as reactive oxygen species (ROS) and reactive nitrogen species (RNS), can be more directly responsible for damage at the cellular level. TNF-α can induce potent oxidizing byproducts directly or indirectly, through membrane signaling inducing free radical-mediated injury. A marked increase in chemically stable oxidative damage products of arachidonic acid (AA) and docosahexaenoic acid (DHA), F2-isoprostanes (F_2_-IP) and F_4_-neuroprostanes (F4-NP), respectively is reported during acute HSV-1 encephalitis in mice (Milatovic et al., 2002). Interestingly, higher levels of F_2_-IP can be observed in infected mice until several weeks after recovery from the acute, symptomatic phase of the infection. Oxidant tissue injury, therefore, is a significant mechanism behind neuronal damage during acute HSV-1 encephalitis.

## Current and Future Therapies to Control Ocular Herpes

Effective management and treatment of ocular herpes remains a challenging task. While antiviral drugs do exist and provide relief from common symptoms, the recurrent use of such drugs can itself cause damaging vision and nephrotoxicity issues (Koganti et al., 2019). Another problem is chronic inflammation that may persist for years after the initial onset of the herpetic eye disease. Usually, a superficial infection of the conjunctiva and corneal epithelium (epithelial keratitis) are easily treatable with existing antivirals and often resolve after the treatment, whereas the infection of the corneal stroma (stromal keratitis) and uveitis remain difficult to treat and often require long-term treatment with antiviral as well as anti-inflammatory drugs such as topical corticosteroids and nucleoside analogs (Liesegang, 1988; Lobo et al., 2019). For superficial keratitis epithelial scraping along with trifluridine drops is routinely prescribed. Oral antivirals, exemplified by acyclovir, valacyclovir and famciclovir constitute the most common treatments for ocular indications as they demonstrate better treatment efficacy and better patient compliance than topical drugs (Koganti et al., 2019). Ganciclovir gel, which is a newer option, also provides good topical efficacy. These drugs are nucleoside analogs that cause termination of HSV-1 DNA synthesis (Koganti et al., 2019). Given the recurrent nature of HSV-1 ocular infection, many patients are prescribed low prophylactic dose of oral acyclovir (Lobo et al., 2019). Since these drugs must be prescribed for very long time, emergence of viral resistance is on the rise. Currently, Foscarnet is the only injectable drug approved for the treatment of acyclovir-resistant mucocutaneous HSV infection (Choong et al., 2010). Drug resistance is hard to control since multitargeted therapy, which has reduced the incidence of drug resistance in other chronic viral diseases, is virtually non-existent against ocular herpes. New drug discoveries that target new viral or host molecules offer hope. These include an HSV-1 gD-targeting aptamer (Yadavalli et al., 2017); innovative use of tranylcypromine to inhibit lysine-specific demethylase 1 (LSD1) which suppresses HSV-1 lytic infection, shedding, and reactivation from latency in animal models (Hill et al., 2014); BX795, which inhibits AKT phosphorylation to block HSV-1 protein synthesis in corneal cells (Jaishankar et al., 2018) and 4-phenylbutyrate (PBA), which reduces ER stress and synergistically increases the efficacy of existing antivirals and by its own, reduces ocular HSV-1 infection (Yadavalli et al., 2020). A couple of above-mentioned drugs such as tranylcypromine and PBA are already approved for other clinical indications, therefore, their approval for treating HSV-1 infection of the eye might be more predictable timewise than the other experimental drugs.

## Conclusion

Many host cell proteins mediate pathophysiological aspects of HSV-1 infection of the cornea. Cells of the cornea can exhibit a complex set of immunopathologies in the form of either epithelial, stromal, or endothelial keratitis. Host response can clear the virus while promoting inflammation and angiogenesis causing tissue damage in the end. While the host proteins activated by the infection may have pro- or antiviral roles, a delicate balance is needed to resolve the disease. Tilting the balance in either direction can be detrimental to corneal health and vision. For the same reason, up-or down-regulating proteins *via* gene therapy may not be a viable option to control the disease but it is likely that dysfunctional or hyperactive proteins can contribute significantly to disease manifestation. Recent information on host proteins in ocular disease manifestation has created unique opportunities for therapeutic development. It is increasingly clear that reducing HPSE activity to normal levels may reduce viral titers as well as inflammation. Fully functional Optineurin, in contrast, may be needed to maintain good ocular as well as neurological health.

## Author Contributions

SS and TV-N wrote and edited the manuscript. Both authors contributed to the article and approved the submitted version.

## Conflict of Interest

The authors declare that the research was conducted in the absence of any commercial or financial relationships that could be construed as a potential conflict of interest.

## Publisher’s Note

All claims expressed in this article are solely those of the authors and do not necessarily represent those of their affiliated organizations, or those of the publisher, the editors and the reviewers. Any product that may be evaluated in this article, or claim that may be made by its manufacturer, is not guaranteed or endorsed by the publisher.
